# The role of glucagon on type 2 diabetes at a glance

**DOI:** 10.1186/1758-5996-6-91

**Published:** 2014-08-24

**Authors:** Amélio F Godoy-Matos

**Affiliations:** Metabolism Unit, Instituto Estadual de Diabetes e Endocrinologia, Rio de Janeiro and Catholic University, Rio de Janeiro, Brazil

**Keywords:** Type 2 diabetes, Hiperglucagonemia, Incretin effect, GLP-1

## Abstract

The opposite effects of insulin and glucagon in fuel homeostasis, the paracrine/endocrine inhibitory effects of insulin on glucagon secretion and the hyperglucagonemia in the pathogenesis of type 2 diabetes (T2D) have long been recognized. Inappropriately increased alpha-cell function importantly contributes to hyperglycemia and reflects the loss of tonic restraint normally exerted by high local concentrations of insulin on alpha-cells, possibly as a result of beta-cell failure and alpha-cell insulin resistance, but additional mechanisms, such as the participation of incretin hormones in this response, have also been suggested. Three classes of drugs already available for clinical use address the abnormalities of glucagon secretion in T2D, namely, the GLP-1 receptor agonists (GLP-1RA), the inhibitors of dipeptidyl peptidase-4 (DPP-4i) and the amylin agonist pramlintide; it has been proposed that the glucagonostatic and insulinotropic effects of GLP-1RA equally contribute to their hypoglycemic efficacy. In this review, the control of glucagon secretion and its participation in T2D pathogenesis are summarized.

## The main players in the control of glucagon secretion

The existence of glucagon was suggested by Murlin et al. [1] in 1923, right after insulin discovery, to explain the precocious hyperglycemic effect of pancreatic extracts, which, according to their hypothesis, was contaminated with a GLUCose AGONist substance. In 1948, Sutherland and de Duve [2] defined the alpha-cells of the islets of Langerhans as the source of glucagon as well as the actions of this hormone stimulating hepatic glycogenolysis and gluconeogenesis in hypoglycemic conditions. In 1959, Unger et al. [3] reported a glucagon radioimmunoassay.

Insulin and glucagon participate in fuel homeostasis, being reciprocally released in response to glycemic oscillations; insulin prevails in the fed state, promoting glucose uptake by its target organs whereas glucagon mobilizes hepatic glucose in the fasting state to ensure the maintenance of normoglycemia [4]. The protective mechanisms against hypoglycemia include suppression of insulin secretion and rise of counterregulatory hormones, especially glucagon and epinephrine.

While it remains ill-defined whether low glucose directly stimulates glucagon release during hypoglycemia, the paracrine/endocrine (interstitium/microcirculation) inhibitory effects of insulin and somastostatin on glucagon secretion are relatively well established [5]. The alpha-cells abundantly express insulin receptors and insulin activates ATP-sensitive K^+^-channels with consequent membrane hyperpolarization [6]. Another mechanism involved in alpha-cell membrane hyperpolarization and glucagon suppression by insulin in rodent islets is the activation of gamma-aminobutyric acid (GABA) receptors through an AKT kinase-dependent pathway [7]. Somatostatin released by delta-cells also activates K^+^-channels in the alpha-cells, besides inhibiting adenylate cyclase activity, cAMP content and protein kinase A (PKA)-stimulated glucagon secretion [5]. Finally, insulin inhibits proglucagon gene transcription, possibly representing a long-term mechanism for regulating alpha-cell function [8]. Beyond this paracrine regulation, glucagon secretion is influenced by autonomic factors originating in brain, where the ventromedial hypothalamus (VMH) is recognized as an important hypoglycemia-sensing area that can modulate glucagon release [9].

Glucagon secretion is also stimulated by the incretin hormone glucose-dependent insulinotropic peptide (GIP) and by epinephrine (although Walker et al. [4] have found that, unlike rat islets, human islets undergo a weak stimulatory effect of adrenaline on glucagon secretion), and is suppressed by leptin [10], amylin [11] and glucagon-like peptide-1 (GLP-1). GLP-1 is an insulinotropic incretin hormone derived from the same gene that encodes glucagon, proglucagon, which originates a preproprotein cleaved into distinct mature peptides depending on the enzyme involved in its post-translactional process; proconvertase (PC)2 in alpha-cells gives rise to glucagon and PC1/3 in intestinal L-cells generates GLP-1 [12]. Interestingly, GLP-1 and PC1/3 immunoreactivity has been detected in subsets of alpha-cells [13, 14], suggesting that glucagon secretion may also be directly regulated by pancreatic GLP-1 [13].

The mechanism by which GLP- 1 decreases glucagon secretion is a matter of debate; reports regarding the expression of GLP-1 receptors in alpha-cells have ranged from absent [15], to very low [16] and to present in 20% of the alpha-cells [17]. Recently, De Marinis et al. reported the expression of GLP-1 receptors in alpha-cells is <0.2% of that in beta-cells and that GLP-1-induced suppression of glucagon release is dependent of PKA and independent of glucose or paracrine effects mediated by insulin or somatostatin [18]. On the other hand, de Heer et al. [19] have previously demonstrated that GLP-1 inhibitory effect on glucagon secretion is mediated by somatostatin acting on somatostatin receptor subtype-2 (SSTR-2).

## Type 2 diabetes and impaired incretin effect

**Figure 1:**
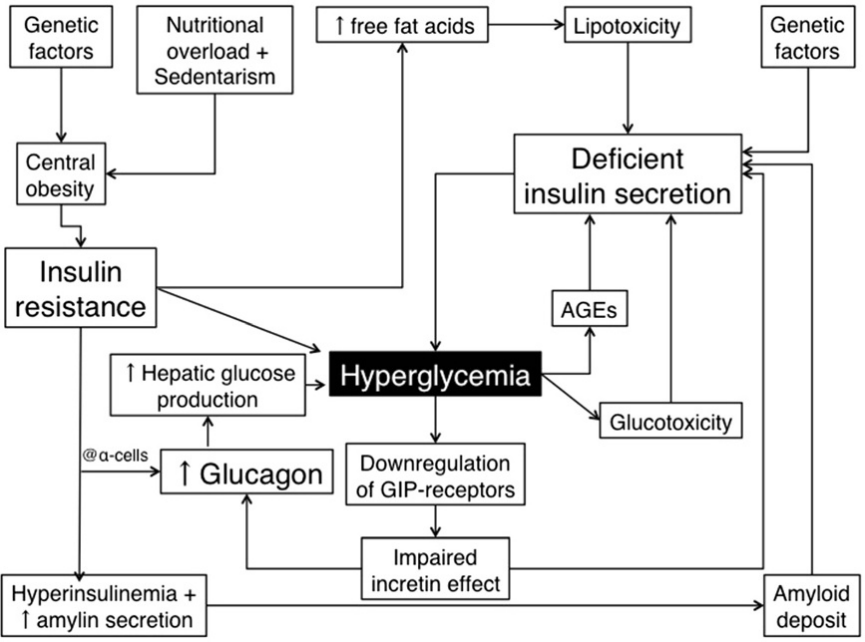
**Main contributors to hyperglycemia in Type 2 diabetes mellitus.** AGE: advanced glycation endproducts.

Type 2 diabetes mellitus (T2D) is characterized by insulin resistance secondary to abnormalities triggered by nutrional overload associated with deficient insulin secretion. The latter condition results from a partial loss of beta-cell mass and beta-cell dysfunction, both influenced by genetic factors and by the chronic exposure of pancreatic islets to glucolipotoxicity, to amylin, the main component of amyloid fibril deposits [20] and to advanced-glycated endproducts [21] (Figure 1).

In 1986, Nauck et al. described a reduced incretin effect in T2D patients, which explains the fact that the insulinotropic incretin hormones GIP and GLP-1 account for <20% of postprandial insulin response [22], while in non-diabetic individuals, both incretin hormones are responsible for 50-70% of the postprandial insulin response [23]. Since then, several studies have addressed the potential mechanisms underlying the reduction in incretin effect in T2D and, to make a long history short, the hypotheses raised by Juris Meier and Michael Nauck are summarized hereafter.These authors propose that reductions in GIP and GLP-1 secretions do not appear to contribute significantly to the loss of incretin effect and that there is a reduction in the insulinotropic action of GIP (whereas GLP-1 action is relatively well preserved) secondarily to a general impairment in beta-cell function. Additionally, hyperglycemia further decreases beta-cell response to GIP because it downregulates its receptor in this cell type (Figure 1).

According to their assumption, the reduction of the incretin effect in T2D patients is an epi-phenomenon of chronic hyperglycemia, irrespective of primary defects in GIP or GLP-1 action [24], which is consistent with the finding of loss of GIP insulinotropic efficacy in patients with diabetes of other etiologies, such as secondary to chronic pancreatitis, monogenic diabetes caused by HNF-1 alpha mutations, and autoimmune diabetes with preserved beta-cell function [25]. Another finding that corroborates the reduction of incretin effect in T2D as a by-product of prolonged hyperglycemia is the improvement of insulin secretion in response to oral compared with intravenous glucose in patients submitted to intensified insulin treatment who significantly improved glycemic control [26].

Even though the reduced incretin effect may be a consequence rather than a causal factor of T2D, some studies have shown that it might be an early sign of impaired glucose metabolism detected before other signs of beta-cell dysfunction are apparent [27]. This is exemplified by the study of Hansen et al., who induced insulin resistance and reduced glucose tolerance in healthy young males (without a family history of diabetes) after daily oral administration of 37.5 mg of prednisolone plus a high-calorie diet and physical inactivity during 12 days and observed a decrease in the incretin effect from 72 ± 5 to 43 ± 7%, while the insulin response to intravenous glucose was capable of completely compensate the impaired insulin sensitivity [28].

## Hyperglucagonemia in T2D development

Although the pathogenesis of T2D is classically focused on insulin resistance and beta-cell dysfunction, the inappropriately increased alpha-cell function and consequent hyperglucagonemia has long been recognised as a contributor to hyperglycemia in diabetic patients, by stimulating hepatic glucose production [29] (Figure 1). Indeed, elevated fasting concentrations of glucagon, as well as impaired glucose-induced glucagon suppression and a disrupted insulin–glucagon interaction in the postprandial period, were described in T2D patients, differently from healthy subjects who present plasmatic glucagon and insulin concentrations inversely related in the postprandial state. The loss of the inverse relationship between these two hormones in T2D patients might be secondary to the observed diminished mass of insulin pulses, and suggests that alterations in the cross-talk between beta- and alpha-cells may underlie hyperglucagonemia [30].

In 1984, Borghi et al. reported that obese non-diabetic insulin-resistant and obese glucose-intolerant subjects already exhibited impaired oral glucose-induced glucagon suppression, even in presence of endogenous hyperinsulinemia and hypothesized that alpha-cells might be resistant to the insulin’s suppressive effect on glucagon secretion [31]. In 2007, Ferrannini et al. [32] measured insulin resistance by the euglycaemic–hyperinsulinaemic clamp in 1,296 non-diabetic individuals and demonstrated that whole-body insulin resistance is independently associated with elevated fasting glucagon concentrations, possibly as a result of alpha-cell insulin resistance. The confirmation of the direct *in vivo* role of insulin signaling in the modulation of alpha-cell function was provided by the conditional alpha-cell specific insulin receptor knock-out (αIRKO) mice, which exhibited up to 50% higher glucose levels compared to control animals in the fed state, as well as hyperglucagonemia [33].

Unger and Orci [34] have recently introduced the term paracrinopathy to designate the loss of tonic restraint normally exerted by a high local concentration of insulin on alpha-cells; beta-cell destruction and beta-cell failure to secrete the first phase of insulin associated with alpha-cells insulin resistance would be the main mechanistic factors in type 1 and type 2 diabetes, respectively.

Besides the lack of inhibitory tone exerted by insulin on glucagon release, other mechanisms have been investigated to explain the inappropriate increased alpha-cell function in T2D. Motivated by the findings of some studies showing that T2D patients, in contrast to their improper glucagon response to oral glucose, are able to suppress glucagon release after an isoglycemic intravenous glucose infusion (IIGI) similarly to non-diabetic subjects, Lund et al. evaluated the role of GIP, GLP-1 and glucagon-like peptide-2 (GLP-2) in this discrepant response. Therefore, plasmatic glucagon concentrations were measured during a 3-h, 50-g oral glucose overload or an IIGI in ten T2D patients; four additional IIGI were performed in which GIP, GLP-1, GLP-2 or a combination of the three were intravenously infused. While no suppression of glucagon was observed during the initial phase of the oral glucose overload, significantly lower plasmatic concentrations of this hormone were observed during the first 30 min of the IIGI. The glucagon response during the IIGI performed with infusion of GIP + GLP-1 + GLP-2 was inappropriate and mimicked the one observed after the oral glucose overload; infusion of GIP alone promoted significant hypersecretion of glucagon, whereas infusion of GLP-1 alone enhanced glucagon suppression during the IIGI. These authors suggested that the improper hyperglucagonemic response to oral glucose could be dependent on the release of the intestinal hormones, especially GIP, which seems to play an important role in this pathophysiological feature [35].

In the pathophysiology of T2D a disbalance in beta-to-alpha-cell ratio, mainly due to beta-cell apoptosis, has also been suggested as a mechanism contributing to a decreased insulin-to-glucagon ratio. However, a new possible mechanism has been put forward in an animal model, suggesting that, under stress demand, beta-cell dedifferentiation to progenitor pluripotent cells takes place. These cells may begin to express, and eventually release, glucagon and somatostatin [36], further contributing to decreased insulin-to-glucagon ratio.

## Addressing glucagon in T2D treatment

Unger and Cherrington [37] have proposed a “glucagonocentric” vision of diabetes pathophysiology, and their arguments for defending this point of view include the following facts: glucagon augments the catabolic processes occurring in the absence of insulin; hyperglucagonemia is present in all forms of poorly controlled diabetes and leptin and somatostatin, known glucagon suppressors, abrogate the catabolic manifestations of diabetes during total insulin deficiency. Perhaps the most astonishing fact had been the finding that glucagon receptor–null mice do not develop diabetes following complete beta-cell destruction [38]. More recently, Omar et al. suggested that the explanation for the absence of hyperglycemia in this mice model may not only be the lack of glucagon effects, but also the presence of high concentrations of fibroblast growth factor 21 (FGF-21) and GLP-1 exhibited by these mice. They demonstrated that the concurrently neutralization of FGF-21 (with a FGF-21 antibody) and GLP-1 (with its antagonist Exendin 9–39) actions resulted in hyperglycemia in those insulin deficient glucagon receptor null mice [39].

Three classes of drugs already available for clinical use address the abnormalities of glucagon secretion in T2D, namely, the GLP-1 receptor agonists (GLP-1RA), the inhibitors of dipeptidyl peptidase-4 (DPP-4i), enzyme that degrades GLP-1 (and other peptides and cytokines) and the amylin agonist pramlintide. The first two classes also exert insulinotropic effects, and the reason why they do not markedly increase plasmatic concentrations of insulin and C-peptide is thought to be in part due to the effect of GLP-1 signaling to lower glycemia, decreasing the stimulus to the beta-cells [40]. Hare et al. [41] suggested that the effect of GLP-1 consists of the improvement of glucose-induced insulin secretion, resulting in rather unchanged absolute secretion rates, while glucagon secretion, which would have been expected to augment with falling glucose concentrations, actually reduces. These authors propose that the glucagonostatic and insulinotropic effects of GLP-1 equally contribute to its hypoglycemic efficacy. In clinical settings, treatment of T2D patients with a DPP-4i, vildagliptin, reduced post-meal glucagon concentrations after 4 weeks of treatment in a manner that the 2-h glucose decrement was significantly related to the 1-h glucagon reduction [42]. In the same way, the GLP-1RA liraglutide significantly reduced the 24-h area under the curve (AUC) of glucagon in comparison to placebo (AUC = 2,179 ± 118 vs 2,371 ± 135, respectively; *P* = 0.037), primarily as a result of a marked reduction in glucagon concentrations after the evening meal [43]. Interestingly, in C-peptide negative Type 1 diabetes patients, liraglutide decreased glucagon after a mixed meal and improved glycemic control while reducing insulin needs [44]. These findings suggest that liraglutide may act inhibiting glucagon regardless of intra-islet insulin, through GLP-1 receptor in alpha-cells or indirectly via somatostatin, as discussed above.

## Conclusions

In summary, the relevance of dysfunctional glucagon secretion to the pathogenesis of diabetes has been widely recognized and, for that reason, targeting glucagon and not only insulin secretion abnormalities in the treatment of T2D has gained increased interest. The well-established actions of GLP-1 as a negative regulator of glucagon and as a positive regulator of insulin and the availabitily of GLP-1RA and DPP-4i provide the opportunity of targeting both main hormones implicated in diabetes pathophysiology. Whether these drugs allow a possible recovery of beta-to-alpha cell mass is a new open avenue for researching.

## Abbreviations

α-IRKO: Alpha-cell specific insulin receptor knock-out
AUC: Area under the curve
DPP-4i: Inhibitors of dipeptidyl peptidase-4
FGF-21: Fibroblast growth factor 21
GABA: Gamma-aminobutyric acid
GIP: Glucose-dependent insulinotropic peptide
GLP-1: Glucagon-like peptide-1
GLP-2: Glucagon-like peptide-2
GLP-1RA: GLP-1 receptor agonists
IIGI: Isoglycemic intravenous glucose infusion
PC: Proconvertase
PKA: Protein kinase A
SSTR-2: Somatostatin receptor subtype-2
T2D: Type 2 diabetes
VMH: Ventromedial hypothalamus
